# On-site generated peroxy acetic acid (PAA) technology reduces *Salmonella* and *Campylobacter* on chicken wings

**DOI:** 10.1016/j.psj.2021.101206

**Published:** 2021-04-21

**Authors:** S. Vaddu, J. Kataria, T.S. Belem, G. Sidhu, A.E. Moller, C. Leone, M. Singh, H. Thippareddi

**Affiliations:** Department of Poultry Science, University of Georgia, Athens, GA 30602

**Keywords:** peroxy acetic acid, *Salmonella*, *Campylobacter*, chicken wings, antimicrobial

## Abstract

Peroxyacetic acid (**PAA**) is a widely used antimicrobial during poultry processing that requires to be shipped in a concentrated solution, stored under hazardous conditions and diluted for use. On-site PAA generation using nonhazardous ingredients can help eliminate transportation and storage issues at the processing plant and reduce the risk of occupational hazards. The objective of the proposed research was to 1) evaluate the efficacy of on-site generated PAA in reducing *Salmonella* and *Campylobacter* populations compared to the commercially available PAA stock solutions and 2) to perform color measurements to evaluate any deviations between treatments. PAA solutions at 50 and 100 ppm were used for treating the chicken wings. Fresh chicken wings (0.45 kg) were inoculated with a cocktail of nalidixic acid resistant *Salmonella* Typhimurium (**STNR**) and gentamicin resistant *Campylobacter coli* (CCGR) and immersed in PAA solutions (50 and 100 ppm) adjusted to pH 8.5 and 10.0 or 10.5, for either 10 s or 60 min. Treated chicken wings were rinsed for 1 min in chilled BPW (100 mL), serially diluted and plated on APC Petrifilm for *Salmonella*, spread plated on Campy-cefex agar supplemented with gentamicin (200 ppm) for *Campylobacter* enumeration. Immersion of chicken wings in 100 ppm PAA for 60 min irrespective of pH levels and PAA solutions resulted in greater microbial reductions (*P* < 0.05) of *Salmonella* by 1.68 and 1.42 log CFU/mL for SaniDate, 1.82 and 1.83 log CFU/mL for OxyFusion (on-site generated). For the same treatments, *Campylobacter* reductions of 1.59 and 1.36 log CFU/mL for SaniDate, 1.63 and 1.71 log CFU/mL for OxyFusion were achieved. The antimicrobial efficiency of PAA was not affected by pH and type of PAA solution. No significant differences (*P* > 0.05) in color were observed between treatments and controls. On-site generated PAA provides poultry processors an effective, safer, and less hazardous alternative to commercially available PAA solutions, ensuring poultry workers’ health and safety.

## INTRODUCTION

Poultry and poultry products represent a popular choice of protein in the United States, with increasingly high consumption rates over the past years (Zhang et al., 2018). Poultry meat has surpassed both pork and beef in per capita consumption in the United States (USDA Economic Research Service, 2020). With this increased consumption of poultry and poultry products, there is a significant concern for contamination, thus impacting public health. *Salmonella* and *Campylobacter* have become major pathogens of concern incriminated in foodborne illnesses related to poultry products. These microorganisms are commensals and can be present in the gastrointestinal tract (**GIT**) of healthy birds and contaminate poultry meat during various stages of processing (Rigby and Pettit, 1980; Stern et al., 1995). In 2018, FoodNet identified 25,606 infections, 5,893 hospitalizations, and 120 deaths due to foodborne sources (Tack et al., 2019). *Salmonella* and *Campylobacter* continue to be major foodborne pathogens resulting in majority of the foodborne illnesses (Chen et al., 2014) and are reported to contribute approximately 73.4% of foodborne infections, 71.7% of hospitalizations and 55% of the total number of deaths due to foodborne illnesses (Tack et al., 2019). There is an increasing evidence of poultry and poultry products being a major contributor to campylobacteriosis in humans (Skirrow, 1982; Altekruse et al., 1999).

The U.S. Department of Agricultures’ Food Safety and inspection Services (**USDA-FSIS**) updated performance standards for *Salmonella* and *Campylobacter* on post chill poultry samples during the production of poultry and poultry meat (USDA, 2016). A maximum of 5 *Salmonella* positive samples out of 51 (9.8%), and a maximum of 8 *Campylobacter* positive samples out of 51 (15.7%) on postchill whole carcasses can be allowed. Additionally, *Salmonella* must be less than 15.4% positive (8 positive samples of 52), and *Campylobacter* must be below 7.7% (4 positive samples of 52) on chicken parts.

Poultry processors have incorporated numerous antimicrobial interventions at various stages of processing (e.g. scalders, inside-outside bird washers, prechillers, main chillers, postchill interventions) to meet the USDA-FSIS performance standards. However, the main chiller and finishing chiller are believed to serve as the most efficient and cost-effective unit operations for antimicrobial application due to the extended or short periods of product exposure, respectively. Additionally, these points of intervention also allow for use of lower or relatively higher PAA concentrations in the chiller water.

Peracetic acid (**PAA**) has been recently used as an antimicrobial in chiller as well as a postchill dip and/ or in a finishing chiller (Kumar et al., 2020). PAA exists in equilibrium with acetic acid, water, and hydrogen peroxide (USNLM, 2011), however the proportion of these mixtures can vary significantly between suppliers, and the regulatory approvals for PAA vary from 50 ppm to 2000 ppm (USDA, 2016). This antimicrobial is effective due to its combined acidic and oxidizing properties (Nagel et al., 2013). PAA has several advantages over other antimicrobials such as chlorine, i.e., it does not form harmful chemical by-products whereas chlorine can form chloroform and bromodichloromethane when in contact with high amounts of organic matter during poultry processing. These by-products are considered human carcinogens and can pose a potential occupational hazard for the plant workers (Occupational Safety and Health Administration, 2017).

PAA is widely used as a postharvest intervention either in prechiller or finishing chiller during poultry processing. Current off-site PAA production requires the concentrated solution be produced, along with addition of stabilizers to minimize decomposition and shipping to the site of usage (poultry processing plants), stored under hazardous conditions, and diluted for use. The vapors and odors of the compounds while mixing to obtain the required concentrations can have irritating properties to the skin and eyes, which might pose considerable levels of occupational hazard to the workers’ health and safety (Budavari, 1996). During storage, concentrated PAA solutions can vaporize and affect employee health if the storage area is not properly ventilated. In addition, stabilizers such as 1-hydroxyethylidene-1,1-diphosphonic acid (**HEDP**) are used to enhance stability of commercially obtained PAA. Although PAA decomposition products, acetic acid, water and oxygen are nontoxic, HEDP residues can be observed on the carcasses after antimicrobial treatment without further washing or processing (EFSA, 2011). While toxic effects of HEDP were not observed, EFSA recommended implementation of HACCP plans to monitor HEDP residues and concentrations on poultry carcasses and environment due to its lack of biodegrading capacity which can pollute the environment when they enter sewage treatment plant (EFSA, 2011). Unlike traditional methods, these concerns can be alleviated by on-site generation of PAA and thus, reduce the occupational hazard risk.

Fletcher (2002) stated that appearance (skin color, meat color and cooked meat pinkness) and texture are the 2 most important quality attributes for poultry meat. Further, Fletcher (2002) stated that consumers consider meat color an important purchase criterion and an indicator of quality and freshness. This is particularly true for broiler and broiler parts that are marketed skin-on, with yellow skin being considered as superior quality product. Some of the antimicrobials used in poultry processing such as chlorine, PAA and others are oxidants and can cause bleaching of the color of the poultry meat, and can lead to unacceptable product, especially in situations where some processors consider it a desirable quality.

PAA solutions without the stabilizers such as HEDP have limited stability and will decompose to constituent parts, acetic acid, water and oxygen. Thus, there is a need to validate the antimicrobial efficacy of the onsite generated PAA with the commercially marketed PAA solutions containing stabilizers. The objectives of the study were to (1) evaluate the efficacy of on-site generated PAA in reducing populations of *Salmonella* and *Campylobacter* in comparison with commercially available PAA stock solutions and (2) evaluate color of poultry meat following different treatments.

## MATERIALS AND METHODS

### Bacterial Strains and Preparation of Inoculum

Nalidixic acid-resistant *Salmonella* Typhimurium (**ST^NA^**) and gentamicin-resistant strain of *Campylobacter coli* (**CC^G^**) were obtained from the U.S. National Poultry Research Center, United States Department of Agriculture, Athens, GA. A loopful of ST^NA^ was transferred from the frozen stock and streaked onto Brilliant Green Sulfa agar (BGS; Difco**,** Sparks, MD) supplemented with 200 ppm of nalidixic acid (Sigma-Aldrich, St. Louis, MO) and incubated for 24 h at 35 ± 1°C. Similarly, a loopful of frozen culture of CC^G^ was streaked onto Campy-cefex agar (Neogen Corporation, Lansing, MI) supplemented with 200 ppm of gentamicin (Sigma-Aldrich, St. Louis, MO) and incubated for 48 h at 42 ± 1°C in a resealable plastic bag flushed with microaerophilic atmosphere (containing 85% N_2_, 10% CO_2_, and 5% O_2_). A cocktail of ST^NA^ and CC^G^ was used to inoculate the chicken wings. An isolated colony of *Salmonella* from BGS agar was used to inoculate 10 mL of Tryptic Soy Broth (TSB; Remel, Lenexa, KS) supplemented with 200 ppm nalidixic acid and incubated for 24 h at 35 ± 1°C. After incubation period, the cultures were centrifuged at 5,500 × *g* for 10 min at 4°C, the pellet was resuspended in 5 mL 0.1% peptone water (**PW**; Difco, Sparks, MD) and centrifuged again. The supernatant was removed and the pellet was resuspended in 0.5 mL of PW.

The CC^G^ was prepared as a lawn on 8 Campy-cefex agar plates, incubated as described previously, and the cells from each plate were harvested using 1 mL buffered peptone water (BPW; Difco, Sparks, MD) per plate and the individual cell suspension from each plate combined. The ST^NA^ cultures were centrifuged at 5500 × g for 10 min at 5°C, and the pellet was resuspended in 5 mL of 0.1% peptone water and this procedure was repeated twice. The 2 cell suspensions (ST^NA^ and CC^G^) were combined and BPW was added to make up a total volume of 250 mL to obtain ca. 6 log CFU/mL of ST^NA^, and 5 log CFU/mL of CC^G^. The cell concentrations of ST^NA^ and CC^G^ were confirmed by direct plating on appropriate media. For each replication, a fresh inoculum was prepared on the day of the experiment.

### PAA Solutions

Two PAA solutions (SaniDate FD Plus; OxyFusion; BioSafe Systems, LLC, East Hartford, CT) were used in this study. SaniDate FD Plus (SaniDate) is a commercially available PAA solution marketed as 19.5-25% peracetic acid, 9.5 to 12% hydrogen peroxide, 36 to 39% acetic acid and HEDP <1%. OxyFusion is an onsite PAA generation technology that combines precursor chemicals (hydrogen peroxide and triacetin) that go through an equilibrium reaction to produce peracetic acid on-demand. Fresh PAA was produced using OxyFusion technology on each day of the experiment.

### Preparation of Antimicrobial Treatments

The antimicrobial treatments evaluated in the study included 50 ppm (0.005%) and 100 ppm (0.01%) peracetic acid (PAA) at 2 pH levels (8.5 and 10 or 10.5), using PAA (SaniDate, OxyFusion). The PAA from SaniDate was acidic as it is an equilibrium mixture with acetic acid. The OxyFusion PAA was in the alkaline range as it does not go through an equilibrium reaction to prevent excess amount of acetic acid and hydrogen peroxide and produces only peroxyacetic acid and some residual NaOH from the reactant mixture.

Stabilized PAA (SaniDate) was obtained from the manufacturer and stored under refrigeration until use. OxyFusion was prepared onsite, chilled in an ice water bath and used within 2 h of production. The pH values of non-pH adjusted OxyFusion PAA solutions were 10 and 10.5 for PAA solutions of 50 and 100 ppm, respectively. For some treatments, the pH of SaniDate was adjusted to pH 8.5 and a high pH (10 and 10.5 for PAA solutions of 50 and 100 ppm, respectively) using sodium hydroxide (NaOH; 10 N, Mallinckrodt Baker Inc., Phillipsburg, NJ). Similarly, the pH of OxyFusion PAA solution was adjusted to 8.5 using sulfuric acid. The PAA solutions for treatment of chicken wings were prepared in plastic buckets (18.9 L; Encore Plastics, Forsyth, GA) using chilled water (≤4°C). The concentration of PAA for each treatment was confirmed using the Peroxychem Titration kit (LaMotte Company, Ocean City, MD) and the pH of the PAA solutions was adjusted by adding either NaOH or H_2_SO_4_; the pH of each treatment was confirmed using a benchtop pH meter (Orion Star A111, Thermo Scientific, Ward Hill, MA). The buckets containing the PAA solutions were placed in ice baskets to maintain temperature (≤4–6°C)

### Inoculation of Chicken Wings

Fresh bone-in, skin-on broiler chicken wing flats (wings) were obtained from a commercial poultry processor on each day of the experiment. For each PAA treatment, chicken wings (ca. 0.45 kg) were placed on sterile stainless-steel rack and inoculated with the ST^NA^ and CC^G^ cocktail by spraying 5 mL on each side and placed in a biological safety cabinet for 15 min to allow bacterial attachment.

### Application of Antimicrobial Treatments

The inoculated chicken wings were transferred to a sterilized stainless steel (SS) wire mesh basket (Model DND-095RND120-C04S, Anysizebasket, York, PA) and immersed in containers with PAA (SaniDate, OxyFusion) with appropriate PAA concentration and solution pH for either 10 s or 60 min. Compressed air (103.4 kPa) was used to provide agitation in the PAA solution using an air compressor (Model D55146 Air Compressor, Dewalt, Towson, MD) at the bottom by the means of 2 concentric circular plastic tubes (ID 0.64 cm; 11.5 and dia 15.4 cm, with 1.6 mm holes drilled at 2.5 cm intervals; McMaster-Carr, Elmherst, IL) affixed to a concentric ceramic plate at the bottom of the buckets to facilitate the uniform distribution of compressed air. All the antimicrobial treatments were prepared fresh on each day of the experiment. Inoculated wings not subjected to any antimicrobial treatment served as a control.

### Bacterial Enumeration

Chicken wings treated with PAA were aseptically transferred into sterile rinse bags (BRB3500; 3M Food Safety, St. Paul, MN) and rinsed with 100 mL of chilled Buffered Peptone Water (BPW; Difco**,** Sparks, MD) supplemented with sodium thiosulfate (0.1%; Acros Organics, Fair Lawn, NJ) for 1 min. Sodium thiosulfate was added to neutralize the effects of residual PAA on the chicken wings. Rinsates for each sample were collected and serially diluted in PW for *Campylobacter* (CC^G^) and PW supplemented with 200 ppm nalidixic acid for *Salmonella* (ST^NA^) enumeration*.* For *Campylobacter* enumeration, serial dilutions were spread-plated on Campy-cefex agar supplemented with 200 ppm gentamicin and the plates were incubated at 42 ± 1°C for 48 h in Ziploc bags under microaerophilic environment. For *Salmonella* (ST^NA^) enumeration, appropriate serial dilutions were plated on Aerobic Plate Count Petrifilm (APC; 3M Food Safety, St. Paul, MN) and incubated at 35 ± 1°C for 24 h. Typical *Campylobacter* and *Salmonella* colonies were enumerated and reported as log CFU/mL.

### Color Measurement

Objective color values (CIE - L*a*b*) were determined on all chicken wings (skin) on control and treated product immediately after the application of treatments, using a Konica Minolta colorimeter (CR400, Konica Minolta Inc., Ramsey, NJ). The colorimeter was calibrated with a reflectance standard white plate supplied by the manufacturer. The L* (lightness), a* (redness), and b* (yellowness) values were recorded by placing the hand-held colorimeter directly in contact with the skin. Triplicate measurements were taken from each chicken wing sample on 3 separate chicken wings for each treatment.

### Experimental Design and Statistical Analyses

A 2 (PAA type; SaniDate or OxyFusion) × 2 (pH values; 8.5 or 10) × 2 (exposure times; 10 s or 60 min) experimental design was used. Three independent replications (n = 3) were performed on 3 different days using fresh chicken wings (0.45 Kg; ca. 8 wing flats/treatment), PAA solutions and fresh bacterial inoculum. Data were analyzed using ANOVA in the GLM of SAS 9.4 (2004; SAS Institute Inc., Cary, NC). Fisher's least significant difference (α = 0.05) was used to separate means of the microbial populations (log CFU/mL).

## RESULTS AND DISCUSSION

The mean pH values of nonadjusted SaniDate PAA solutions used for the preparation of PAA treatments were 4.7 ± 0.4 and 4.3 ± 0.2 for 50 and 100 ppm PAA concentrations, respectively. The pH of OxyFusion PAA solutions (nonadjusted) were 10.0 ± 0.3 and 10.5 ± 0.1 for 50 and 100 ppm, respectively. Typical PAA concentrations used in the pre- and main chiller were used to replicate industry practices to evaluate the efficacy of on-site generated PAA in reducing *Salmonella* and *Campylobacter* populations. The temperatures of PAA solutions were maintained between 4 and 6°C throughout the study.

The efficacy of OxyFusion and SaniDate PAA in reducing *Salmonella* and *Campylobacter* populations on chicken wings was evaluated at 2 values: 1) pH 8.5 representing the pKa of PAA and the common pH poultry processors adjust the PAA solution in the main chiller and 2) natural pH of the OxyFusion (ca. pH 10 for the 50 ppm and pH 10.5 for the 100 ppm solution). Adjusting the pH toward the alkaline range improves moisture gain by the broilers during extended exposure (≥45 min) compared to extended exposure to the acidic pH (nonpH adjusted PAA) of commercially available PAA mixtures (PAA, acetic acid and hydrogen peroxide, along with stabilizers).

Poultry processors have incorporated antimicrobial interventions at numerous steps in the first and second processing stages and further cut-up processes during slaughter and processing of broilers. However, the treatment time varies significantly between each of the antimicrobial interventions from 5 s to 60 min, with short exposure times where either the carcasses are immersed or sprayed after picking (postpick immersion), after evisceration (online reprocessing; OLR), post-OLR dip, chillers (premain and postchill immersion) or for chicken parts subsequent to parts cup-up process. While it is optimal to use broiler carcasses to evaluate the potential reductions in foodborne pathogen (*Salmonella* and/or *Campylobacter*) populations achieved at each of these antimicrobial interventions, inoculation of broiler carcasses and their treatment can be unwieldy, if not impossible. An alternate to the use of broiler carcasses for such purposes would be to use chicken parts that have skin on them as the reductions in microorganisms achieved on chicken parts with skin-on parts (such as wing flats, drums, etc.) are lower than the reductions achieved on boneless, skin-less parts (breast fillets, tenders, etc.). As majority of the surface of the chicken wing flats is covered with skin, these can serve as worst-case scenario in terms of microbial reductions achieved by antimicrobial interventions. As such, we have extensively used chicken wing flats as a “model” for broiler carcasses as well as for parts to evaluate the minimal reductions in foodborne pathogens that can be achieved by the antimicrobial interventions. In this study, we evaluated reductions in *Salmonella* and *Campylobacter* populations achieved by the various PAA treatment combinations (type, concentration and pH) at 2 PAA exposure times of 15 s to represent treatment of chicken parts and 60 min to represent treatment of carcasses during chilling.

Inoculation of chicken wings resulted in *Salmonella* and *Campylobacter* populations of 6.0 and 5.11 log CFU/mL, respectively (Table 1 & 2). Immersion of chicken wings in SaniDate PAA solutions of 50 and 100 ppm for 10 s resulted in *Salmonella* reductions of 0.57 and 0.96 log CFU/mL, at pH 8.5, respectively (Table 1). Likewise, immersion of chicken wings in OxyFusion PAA solutions of 50 and 100 ppm resulted in *Salmonella* reductions of 0.75 and 0.92 log CFU/mL at pH 8.5, respectively. Greater reductions in *Salmonella* populations were observed after immersion of inoculated chicken wings in PAA solutions (SaniDate or OxyFusion) of 50 ppm for longer period (60 min), with overall reductions of 1.48 and 1.78 log CFU/mL at pH 8.5, respectively. PAA solution pH did not affect (*P* ≤ 0.05) the *Salmonella* reductions observed on chicken wings subsequent to immersion in PAA solutions (within the same concentration and exposure time), regardless of the type (SaniDate, OxyFusion). Previously, Kataria et al. (2020) reported no differences in the reductions of *Salmonella* and *Campylobacter* when chicken wings were immersed in PAA adjusted to different pH values (8.2, 10 and 11). Increasing the PAA concentrations (from 50 ppm to 100 ppm) and treated for 10 s showed similar *Salmonella* reductions (*P* > 0.05). Kumar et al. (2020) reported similar *Salmonella* reductions of 0.44, 0.57 and 1.01 log CFU/mL following immersion treatment with 100 ppm PAA solutions for 4, 10 and 30 s, respectively on chicken breast fillets (boneless, skinless). Bauermeister et al. (2008b) reported *Salmonella* reductions of 0.76 and 1.05 log CFU/mL following a 60 min immersion treatment with PAA at 25 and 100 ppm. In general, *Salmonella* reductions were greater (*P* ≤ 0.05) with increasing PAA concentration (50 ppm vs. 100 ppm) and when chicken wings were immersed for longer time (10 s vs. 60 min). Within each of the exposure time (10 s or 60 min) and PAA concentrations (50 or 100 ppm), OxyFusion PAA solutions showed similar *Salmonella* reductions as commercially available, stabilized PAA (SaniDate).

**Table 1:** *Salmonella* populations (mean log CFU/mL ± S.D.) on inoculated chicken wings after exposure to various concentrations of peroxy acetic acid from different sources (SaniDate FD and OxyFusion [generated onsite] and adjusted to various pH values.

Conc. (ppm)	Exposure time	SaniDate FD		OxyFusion	
		pH 8.5	High pH§	pH 8.5	High pH
0 ppm (Control†)	-	6.00 ± 0.12^m^			
50 ppm	10 s	5.43 ± 0.14^axm^	5.44 ± 0.29^axm^	5.25 ± 0.28^ax^	5.34 ± 0.42^ax^
	60 min	4.52 ± 0.41^ayz^	4.57 ± 0.40^ay^	4.22 ± 0.59^ay^	4.44 ± 0.41^ay^
100 ppm	10 s	5.04 ± 0.29^axy^	5.25 ± 0.47^ax^	5.08 ± 0.31^ax^	5.19 ± 0.18^ax^
	60 min	4.32 ± 0.19^az^	4.58 ± 0.26^ay^	4.18 ± 0.55^ay^	4.17 ± 0.41^ay^

Same superscripts (^ab^) within the same row indicate no significant differences (*P* > 0.05); same superscripts (^xy^) within the same column indicate no significant differences (*P* > 0.05).

§ High pH – the pH of OxyFusion was 10.0 and 10.5 for 50 and 100 ppm solutions, respectively. SaniDate pH was adjusted to the same pH values as OxyFusion pH values for each concentration.

† Inoculated chicken wings not exposed to PAA solutions for either 10 s or 60 min.

**Table 2:** *Campylobacter* populations (mean log CFU/mL ± S.D.) on inoculated chicken wings after exposure to various concentrations of peroxy acetic acid from different sources (SaniDate FD and OxyFusion [generated onsite] and adjusted to various pH values.

Conc. (ppm)	Exposure time	SaniDate FD		OxyFusion	
		pH 8.5	High pH§	pH 8.5	High pH
0 ppm (Control†)	-	5.11 ± 0.21^by^			
50 ppm	10 s	4.30 ± 0.82^abxy^	4.49 ± 0.58^abxy^	4.15 ± 0.9^abxy^	4.32 ± 0.61^abxy^
	60 min	3.41 ± 1.26^ax^	3.97 ± 0.60^abxy^	3.50 ± 1.10^ax^	3.80 ± 0.57^abxy^
100 ppm	10 s	4.09 ± 0.58^abxy^	4.37 ± 0.93^abxy^	4.19 ± 0.90^abxy^	4.35 ± 0.82^abxy^
	60 min	3.52 ± 0.77^ax^	3.75 ± 0.77^abxy^	3.48 ± 0.76^ax^	3.40 ± 0.87^ax^

Same superscripts (^ab^) within the same row indicate no significant differences (*P*> 0.05); same superscripts (^xy^) within the same column indicate no significant differences (*P* > 0.05).

§ High pH – the pH of OxyFusion was 10.0 and 10.5 for 50 and 100 ppm solutions, respectively. SaniDate pH was adjusted to the same pH values as OxyFusion pH values for each concentration.

† Inoculated chicken wings not exposed to PAA solutions for either 10 s or 60 min.

Immersion of chicken wings in SaniDate PAA 50 and 100 ppm (pH 8.5) for 10 s resulted in *Campylobacter* reductions of 0.81 and 1.02 log CFU/mL, respectively (Table 1). Likewise, immersion of chicken wings in OxyFusion PAA 50 and 100 ppm (pH 8.5) resulted in *Campylobacter* reductions of 0.96 and 0.92 log CFU/mL, respectively. Greater reductions in *Campylobacter* populations were observed after immersion in PAA solutions (both SaniDate, OxyFusion) of 50 and 100 ppm (pH 8.5) for longer period (60 min), with reductions of 1.69 and 1.59, 1.31 and 1.71 log CFU/mL, respectively. In general, immersion of inoculated chicken wings in PAA solutions of increasing concentrations resulted in numerically greater *Campylobacter* reductions (except for SaniDate, pH 8.5 with exposure time of 60 min), although statistically similar (*P* > 0.05), within each of the PAA solution pH treatments, regardless of the exposure time (10 s or 60 min; Table 2). Within each of the exposure time (10 s or 60 min), the *Campylobacter* populations were similar for both the pH values of PAA solutions within each PAA concentration (50, 100 ppm).

Kumar et al. (2020) reported reduction in *Campylobacter* population of 0.2, 0.4 and 0.8 log CFU/mL on chicken breast fillets (skinless, boneless) when immersed in PAA solutions of 100 ppm for 4, 10 and 30 s, respectively. Smith et al. (2015) reported similar reductions in *Campylobacter* populations (ca. 0.7 and 1.4 log CFU/mL) on immersion of broiler carcasses in PAA solutions of 100 ppm and 200 ppm for 60 s, respectively.

Poultry skin color is a major attribute and several processors market the boilers and related products based on the skin color as “golden yellow”, equating it to improved quality and freshness of the broilers. Poultry skin color can range from cream to yellow and is primarily influenced by diet such as carotenoids and other pigments which are deposited in the epidermis (Fletcher, 1992). Poultry processing conditions, especially higher scalding temperatures (Petracci et al., 2010) and the use of oxidizers such as chlorine and PAA (depending on the concentration, exposure time and temperature) can affect poultry skin color.

Immersion of chicken wings in PAA solutions, regardless of the preparation method of PAA, exposure time and pH did not affect the L* values compared to the nontreated control (Table 3). Similarly, Bauermeister et al. (2008b) reported that immersion of broilers in PAA solutions up to 200 ppm for 60 min did not affect the L* values (*P* > 0.05) of chicken skin. However, the authors reported lower L* value (*P* ≤ 0.05) for carcasses treated with 30 ppm of chlorine compared to nontreated control. Izat et al. (1990) reported discoloration of poultry skin when broilers were immersed in lactic acid (1 or 2%) for periods exceeding 60 s. Thiessen et al. (1984) reported broiler skin as being lighter in color compared to the controls when broiler carcasses were treated with different concentrations of chlorine dioxide (0.5–1.39 mg/L). In our study, we used chicken wings from a commercial processor that uses hard scalding (higher temperature of scald water) and does not supplement color generating pigments (in the feed) during bird growth to develop yellow color. Thus, the lack of change in chicken skin color may be due to the lower L* and b* values of the chicken wings resulting from the birds that do not show pigmentation and the use of hard scalding process. Del Rio et al. (2007) reported no difference in color, smell and overall acceptability of chicken legs dipped in 220 ppm PAA for 15 min than untreated controls.

**Table 3:** Instrumental color (L*; mean ± S.D.) of chicken wings after exposure to various concentrations of peroxy acetic acid from different sources (SaniDate FD and OxyFusion [generated onsite] and adjusted to various pH values.

Conc. (ppm)	Exposure time	SaniDate FD		OxyFusion	
		pH 8.5	High pH§	pH 8.5	High pH
0 ppm (Control†)	-	78.20 ± 0.78			
50 ppm	10 s	78.65 ± 1.58	78.23 ± 1.71	77.90 ± 1.34	77.79 ± 1.39
	60 min	78.40 ± 0.83	78.56 ± 0.64	78.48 ± 1.39	78.35 ± 0.91
100 ppm	10 s	78.82 ± 1.34	79.10 ± 1.39	78.63 ± 1.14	77.74 ± .76
	60 min	78.15 ± 2.6	78.46 ± 2.73	79.02 ± 1.05	79.00 ± 0.88

Same superscripts (^ab^) within the same row indicate no significant differences (*P* > 0.05); same superscripts (^xy^) within the same column indicate no significant differences (*P* > 0.05).

§ High pH – the pH of OxyFusion was 10.0 and 10.5 for 50 and 100 ppm solutions, respectively. SaniDate pH was adjusted to the same pH values as OxyFusion pH values for each concentration.

† Inoculated chicken wings not exposed to PAA solutions for either 10 s or 60 min.

Immersion of chicken wings in PAA solutions did not affect (*P* > 0.05) the a* values compared to the controls (Table 4), except for the OxyFusion PAA pH 10, 100 ppm applied for 60 min treatment, which had lower a* value (*P* < 0.05). Castaneda et al. (2005) reported an a* value of 4.84 for carcasses from control birds (nonpigment supplemented in feed) compared to a* values of 1.83 for carcasses from birds fed pigment augmented feed (natural low or high; US- or Mexican blend).

**Table 4:** Instrumental color (a*; mean ± S.D.) of chicken wings after exposure to various concentrations of peroxy acetic acid from different sources (SaniDate FD and OxyFusion [generated onsite] and adjusted to various pH values.

Conc. (ppm)	Exposure time	SaniDate FD		OxyFusion	
		pH 8.5	High pH§	pH 8.5	High pH
0 ppm (Control†)	-	3.08 ± 2.66^axm^			
50 ppm	10 s	3.23 ± 1.03^ax^	3.81 ± 2.01^axy^	2.45 ± 2.54^ax^	2.38 ± 0.76^ax^
	60 min	1.75 ± 1.31^ax^	2.23 ± 0.84^ax^	2.54 ± 2.38^ax^	2.56 ± 1.38^ax^
100 ppm	10 s	3.28 ± 2.19^ax^	4.10 ± 1.79^amy^	3.17 ± 1.68^ax^	2.34 ± 1.99^ax^
	60 min	2.42 ± 1.58^ax^	2.06 ± 1.16^abx^	1.83 ± 2.31^abx^	0.35 ± 2.25^by^

Same superscripts (^ab^) within the same row indicate no significant differences (*P* > 0.05); same superscripts (^xy^) within the same column indicate no significant differences (*P* > 0.05).

§ High pH – the pH of OxyFusion was 10.0 and 10.5 for 50 and 100 ppm solutions, respectively. SaniDate pH was adjusted to the same pH values as OxyFusion pH values for each concentration.

† Inoculated chicken wings not exposed to PAA solutions for either 10 s or 60 min.

**Table 5:** Instrumental color (b*) of chicken wings after exposure to various concentrations of peroxy acetic acid from different sources (SaniDate FD and OxyFusion [generated onsite] and adjusted to various pH values.

Conc.	Exposure time	SaniDate FD		OxyFusion	
		pH 8.5	High pH§	pH 8.5	High pH
0 ppm (Control†)	-	1.63 ± 1.32^by^			
50 ppm	10 s	1.91 ± 1.47^axy^	1.81 ± 1.87^axy^	1.53 ± 1.43^axy^	1.76 ± 1.33^axy^
	60 min	2.71± 1.04^axy^	2.73 ± 1.48^axy^	2.14 ± 1.94^axy^	2.35 ± 2.18^axy^
100 ppm	10 s	2.17 ± 1.90^abxy^	2.53 ± 1.94^axy^	0.77 ± 1.27^bxy^	1.76 ± 1.33^abxy^
	60 min	2.72 ± 1.01^axy^	3.33 ± 2.29^ax^	1.05 ± 1.37^bxy^	2.39 ± 1.47^abxy^

Same superscripts (^ab^) within the same row indicate no significant differences (*P* > 0.05); same superscripts (^xy^) within the same column indicate no significant differences (*P* > 0.05).

§ High pH – the pH of OxyFusion was 10.0 and 10.5 for 50 and 100 ppm solutions, respectively. SaniDate pH was adjusted to the same pH values as OxyFusion pH values for each concentration.

† Inoculated chicken wings not exposed to PAA solutions for either 10 s or 60 min.

Immersion of chicken wings in PAA solution (SaniDate) showed an increase in the b* value with higher concentrations and longer periods of exposure for solutions adjusted to pH 8.5 and the higher pH (10 or 10.5). However, exposure of chicken wings to OxyFusion PAA solution adjusted to pH 8.5 showed lower b* values, although they were statistically similar (*P* > 0.05). In contrast, Bauermeister et al. (2008b) reported a lower b* values for broiler skin color on exposure to up to 200 ppm PAA solutions. This difference in the b* values from the current study and Bauermeister et al. (2008a) could be due to the pH values of the PAA solutions, with the reported pH of the PAA solutions being 4.5, whereas the pH of the PAA solutions was adjusted to 8.5 or 10.0 in the current study.

## CONCLUSIONS

Immersion of inoculated chicken wings in PAA solutions reduced *Salmonella* and *Campylobacter* populations, with longer exposure times (10 s vs. 60 min) resulting in greater reductions. Higher concentrations of PAA solutions resulted in greater reductions on inoculated chicken wings resulted in greater reductions of *Salmonella* and *Campylobacter*. The *Salmonella* and *Campylobacter* reductions achieved on chicken wings on exposure to PAA were similar, regardless of the type of PAA (SaniDate FD or OxyFusion). Some poultry processors are currently adjusting the chiller water containing PAA, regardless of the PAA concentration to pH ≥ 8.5, and in some cases, up to 10.0. In such cases, use of on-site generated PAA (OxyFusion) which results in a natural pH of 10.0 or 10.5 might be more appropriate for use. Minimal changes in the instrumental color of the chicken wings (skin) was observed on exposure to PAA solutions (SaniDate FD and OxyFusion), regardless of the pH, concentration and exposure time. On-site generation of PAA (OxyFusion) can be used by poultry processors and can minimize the occupational risk of transporting and storage of highly oxidative chemical (PAA solution in equilibrium with hydrogen peroxide and acetic acid).

## ACKNOWLEDGMENTS

The authors wish to acknowledge Dr. Estefania Novoa Rama, Ms. Anju Singh and Mr. Shakir Jones for their assistance with the project. The authors thank Biosafe Systems LLC for the funding and Dr. Vijay Choppakatla and Mr. Jeff Madewell for their technical expertise.

## DISCLOSURES

The authors declare no conflicts of interest.
